# New Medical Journal

**Published:** 1859-12

**Authors:** 


*New Medical Journal.*—We have received No. 1 of the *Chicago Medical Examiner*, a monthly journal, edited by Prof. N. S. Davis, and Dr. E. A. Steele, of the new school. The number is neatly issued, and is full of matter of interest. We exchange with pleasure.

